# 4-Methoxy­anilinium bromide

**DOI:** 10.1107/S160053680903503X

**Published:** 2009-09-05

**Authors:** Xue-qun Fu

**Affiliations:** a Ordered Matter Science Research Center, Southeast University, Nanjing 210096, People’s Republic of China

## Abstract

The title compound, C_7_H_10_NO^+^·Br^−^, consists of almost planar 4-methoxy­anilinium cations, wherein the O atom lies 0.049 (3) Å out of the plane formed by the non-H atoms, and a Br^−^ anion. Strong N—H⋯Br and N—H⋯(Br,Br) hydrogen bonding contributes to the stability of the crystal structure and links the cations and anions into a three-dimensional network.

## Related literature

For background to dielectric–ferroelectric materials, see: Hang *et al.* (2009 ▶); Li *et al.* (2008 ▶). For related structures, see: Tan *et al.* (2006 ▶); Soumhi *et al.* (2006 ▶); Ben Amor *et al.* (1995 ▶).
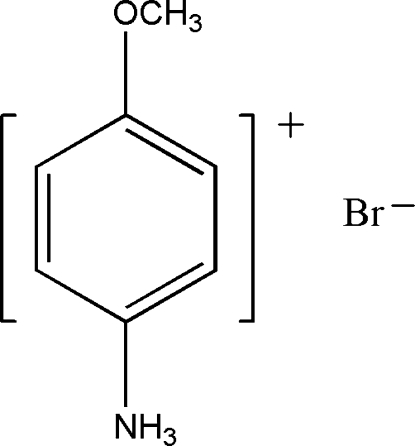


## Experimental

### 

#### Crystal data

C_7_H_10_NO^+^·Br^−^
*M* *_r_* = 204.07
Orthorhombic, 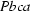

*a* = 8.9779 (18) Å
*b* = 8.6978 (17) Å
*c* = 22.132 (4) Å
*V* = 1728.2 (6) Å^3^
*Z* = 8
Mo *K*α radiation
μ = 4.69 mm^−1^
*T* = 298 K
0.20 × 0.20 × 0.20 mm

#### Data collection

Rigaku SCXmini diffractometer
Absorption correction: multi-scan (*CrystalClear*; Rigaku, 2005 ▶) *T*_min_ = 0.391, *T*_max_ = 0.391
16334 measured reflections
1985 independent reflections
1276 reflections with *I* > 2σ(*I*)
*R*_int_ = 0.075

#### Refinement

*R*[*F* ^2^ > 2σ(*F* ^2^)] = 0.043
*wR*(*F* ^2^) = 0.100
*S* = 1.12
1985 reflections
93 parameters
H-atom parameters constrained
Δρ_max_ = 0.39 e Å^−3^
Δρ_min_ = −0.33 e Å^−3^

### 

Data collection: *CrystalClear* (Rigaku, 2005 ▶); cell refinement: *CrystalClear*; data reduction: *CrystalClear*; program(s) used to solve structure: *SHELXS97* (Sheldrick, 2008 ▶); program(s) used to refine structure: *SHELXL97* (Sheldrick, 2008 ▶); molecular graphics: *SHELXTL* (Sheldrick, 2008 ▶); software used to prepare material for publication: *PRPKAPPA* (Ferguson, 1999 ▶).

## Supplementary Material

Crystal structure: contains datablocks I, global. DOI: 10.1107/S160053680903503X/pv2194sup1.cif

Structure factors: contains datablocks I. DOI: 10.1107/S160053680903503X/pv2194Isup2.hkl

Additional supplementary materials:  crystallographic information; 3D view; checkCIF report

## Figures and Tables

**Table 1:** Hydrogen-bond geometry (Å, °)

*D*—H⋯*A*	*D*—H	H⋯*A*	*D*⋯*A*	*D*—H⋯*A*
N1—H1*A*⋯Br1	0.89	2.52	3.409 (3)	177
N1—H1*B*⋯Br1^i^	0.89	2.55	3.314 (3)	145
N1—H1*B*⋯Br1^ii^	0.89	3.00	3.430 (3)	112
N1—H1*C*⋯Br1^iii^	0.89	2.57	3.300 (3)	140

Symmetry codes: (i) 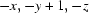; (ii) 
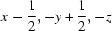
; (iii) 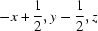.

**Fig. 1.**
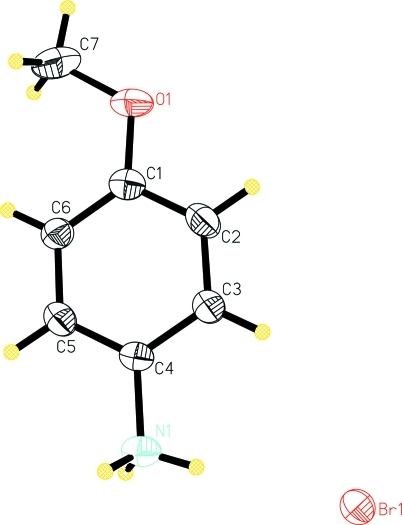
The molecular structure of the title compound, with the atomic numbering scheme. Displacement ellipsoids are drawn at 30% probability level.

**Fig. 2.**
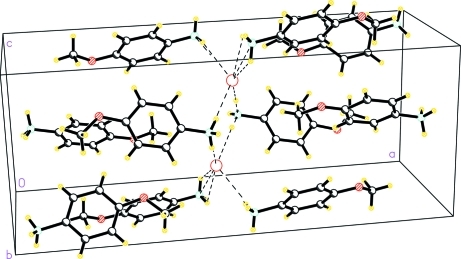
A view of the packing of the title compound; dashed lines indicate hydrogen bonds.

## supplementary crystallographic information

### Comment

This study is a part of systematic investigation of dielectric-ferroelectric materials, including organic ligands (Li *et al.*, 2008), metal-organic coordination compounds (Hang *et al.*, 2009) and organic-inorganic hybrids. The title compound, 4-methoxyanilinium bromide, (I), has no dielectric disuniform from 80 K to 450 K, (m.p. 458–459 K). In this article, the crystal structure of (I) has been presented.

The asymmetric unit of the title compound is built up from an almost planar 4-methoxybenzenamine cation wherein O1 lies 0.049 (3) Å out of the plane formed by its non-hydrogen atoms and a Br^-^ anion (Fig. 1). The strong N—H···Br hydrogen bonding (N···Br distances 3.300 (3)–3.430 (3) Å) contribute to the stability of the crystal structure and lead the cations and anions to tridimensional network (Fig 2). The crystal structures containing 4-methoxybenzenamine cation have been reported (Tan *et al.*, 2006; Soumhi *et al.*, 2006; Ben Amor *et al.*, 1995).

### Experimental

Single crystals of 4-methoxyanilinium bromide were prepared by slow evaporation at room temperature of an ethanol solution of 4-methoxybenzenamine and hydrobromic acid.

### Refinement

Positional parameters of all the H atoms were calculated geometrically and were allowed to ride on the C atoms to which they are bonded, with *U*_iso_(H) = 1.2*U*_eq_(C).

### Crystal data

**Table:**

C_7_H_10_NO^+^·Br^−^	*F*(000) = 816
*M**_r_* = 204.07	*D*_x_ = 1.569 Mg m^−^^3^
Orthorhombic, *P**b**c**a*	Mo *K*α radiation, λ = 0.71073 Å
Hall symbol: -P 2ac 2ab	Cell parameters from 6139 reflections
*a* = 8.9779 (18) Å	θ = 3.3–27.7°
*b* = 8.6978 (17) Å	µ = 4.69 mm^−^^1^
*c* = 22.132 (4) Å	*T* = 298 K
*V* = 1728.2 (6) Å^3^	Prism, colourless
*Z* = 8	0.20 × 0.20 × 0.20 mm

### Data collection

**Table:**

Rigaku SCXmini diffractometer	1985 independent reflections
Radiation source: fine-focus sealed tube	1276 reflections with *I* > 2σ(*I*)
graphite	*R*_int_ = 0.075
Detector resolution: 13.6612 pixels mm^-1^	θ_max_ = 27.5°, θ_min_ = 3.4°
ω scans	*h* = −11→11
Absorption correction: multi-scan (*CrystalClear*; Rigaku, 2005)	*k* = −11→11
*T*_min_ = 0.391, *T*_max_ = 0.391	*l* = −28→28
16334 measured reflections	

### Refinement

**Table:**

Refinement on *F*^2^	Secondary atom site location: difference Fourier map
Least-squares matrix: full	Hydrogen site location: inferred from neighbouring sites
*R*[*F*^2^ > 2σ(*F*^2^)] = 0.043	H-atom parameters constrained
*wR*(*F*^2^) = 0.100	*w* = 1/[σ^2^(*F*_o_^2^) + (0.0263*P*)^2^ + 1.3429*P*] where *P* = (*F*_o_^2^ + 2*F*_c_^2^)/3
*S* = 1.12	(Δ/σ)_max_ = 0.001
1985 reflections	Δρ_max_ = 0.39 e Å^−^^3^
93 parameters	Δρ_min_ = −0.33 e Å^−^^3^
0 restraints	Extinction correction: *SHELXL97* (Sheldrick, 2008), Fc^*^=kFc[1+0.001xFc^2^λ^3^/sin(2θ)]^-1/4^
Primary atom site location: structure-invariant direct methods	Extinction coefficient: 0.0232 (9)

### Special details

**Table:**

Geometry. All esds (except the esd in the dihedral angle between two l.s. planes) are estimated using the full covariance matrix. The cell esds are taken into account individually in the estimation of esds in distances, angles and torsion angles; correlations between esds in cell parameters are only used when they are defined by crystal symmetry. An approximate (isotropic) treatment of cell esds is used for estimating esds involving l.s. planes.
Refinement. Refinement of *F*^2^ against ALL reflections. The weighted *R*-factor *wR* and goodness of fit *S* are based on *F*^2^, conventional *R*-factors *R* are based on *F*, with *F* set to zero for negative *F*^2^. The threshold expression of *F*^2^ > σ(*F*^2^) is used only for calculating *R*-factors(gt) *etc*. and is not relevant to the choice of reflections for refinement. *R*-factors based on *F*^2^ are statistically about twice as large as those based on *F*, and *R*- factors based on ALL data will be even larger.

### Fractional atomic coordinates and isotropic or equivalent isotropic displacement parameters (Å^2^)

**Table:**

	*x*	*y*	*z*	*U*_iso_*/*U*_eq_	
O1	0.1517 (3)	0.3060 (4)	0.29591 (13)	0.0721 (9)	
N1	0.0282 (4)	0.2527 (4)	0.05037 (14)	0.0548 (9)	
H1A	0.0842	0.3201	0.0305	0.066*	
H1B	−0.0676	0.2748	0.0447	0.066*	
H1C	0.0469	0.1583	0.0369	0.066*	
C1	0.1170 (5)	0.2823 (5)	0.23712 (18)	0.0497 (10)	
C2	0.1934 (5)	0.3727 (5)	0.1965 (2)	0.0650 (12)	
H2A	0.2650	0.4412	0.2105	0.078*	
C3	0.1653 (5)	0.3630 (5)	0.13576 (18)	0.0596 (11)	
H3A	0.2166	0.4258	0.1088	0.071*	
C4	0.0621 (4)	0.2612 (4)	0.11502 (17)	0.0427 (9)	
C5	−0.0123 (4)	0.1673 (5)	0.15443 (17)	0.0538 (10)	
H5A	−0.0808	0.0958	0.1401	0.065*	
C6	0.0153 (5)	0.1797 (5)	0.21591 (17)	0.0533 (11)	
H6A	−0.0363	0.1173	0.2429	0.064*	
C7	0.0851 (7)	0.2069 (6)	0.3394 (2)	0.0914 (17)	
H7A	0.1184	0.2351	0.3791	0.137*	
H7B	0.1134	0.1025	0.3312	0.137*	
H7C	−0.0213	0.2162	0.3373	0.137*	
Br1	0.25218 (4)	0.51025 (4)	−0.021898 (16)	0.0495 (2)	

### Atomic displacement parameters (Å^2^)

**Table:**

	*U*^11^	*U*^22^	*U*^33^	*U*^12^	*U*^13^	*U*^23^
O1	0.084 (2)	0.088 (2)	0.0449 (17)	0.0026 (19)	−0.0164 (17)	−0.0048 (17)
N1	0.057 (2)	0.059 (2)	0.0480 (19)	0.0099 (16)	−0.0074 (17)	−0.0047 (15)
C1	0.048 (2)	0.053 (2)	0.048 (2)	0.011 (2)	−0.0035 (19)	−0.006 (2)
C2	0.067 (3)	0.068 (3)	0.060 (3)	−0.021 (2)	−0.008 (2)	−0.009 (2)
C3	0.063 (3)	0.066 (3)	0.050 (3)	−0.017 (2)	−0.001 (2)	−0.001 (2)
C4	0.042 (2)	0.045 (2)	0.041 (2)	0.0082 (17)	−0.0029 (17)	−0.0057 (17)
C5	0.049 (2)	0.054 (3)	0.058 (3)	−0.005 (2)	−0.007 (2)	−0.006 (2)
C6	0.051 (3)	0.061 (3)	0.048 (2)	−0.004 (2)	0.001 (2)	0.0055 (19)
C7	0.107 (4)	0.120 (5)	0.047 (3)	0.011 (4)	−0.007 (3)	0.012 (3)
Br1	0.0493 (3)	0.0461 (3)	0.0531 (3)	0.00163 (19)	−0.0010 (2)	−0.00423 (18)

### Geometric parameters (Å, °)

**Table:**

O1—C1	1.354 (5)	C3—C4	1.361 (5)
O1—C7	1.424 (6)	C3—H3A	0.9300
N1—C4	1.465 (5)	C4—C5	1.369 (5)
N1—H1A	0.8893	C5—C6	1.387 (5)
N1—H1B	0.8903	C5—H5A	0.9300
N1—H1C	0.8899	C6—H6A	0.9300
C1—C6	1.360 (6)	C7—H7A	0.9600
C1—C2	1.377 (6)	C7—H7B	0.9600
C2—C3	1.370 (5)	C7—H7C	0.9600
C2—H2A	0.9300		
			
C1—O1—C7	117.5 (4)	C3—C4—C5	120.4 (4)
C4—N1—H1A	109.5	C3—C4—N1	120.3 (4)
C4—N1—H1B	109.2	C5—C4—N1	119.4 (3)
H1A—N1—H1B	109.5	C4—C5—C6	119.4 (4)
C4—N1—H1C	109.6	C4—C5—H5A	120.3
H1A—N1—H1C	109.6	C6—C5—H5A	120.3
H1B—N1—H1C	109.5	C1—C6—C5	120.6 (4)
O1—C1—C6	125.9 (4)	C1—C6—H6A	119.7
O1—C1—C2	115.2 (4)	C5—C6—H6A	119.7
C6—C1—C2	118.9 (4)	O1—C7—H7A	109.5
C3—C2—C1	120.9 (4)	O1—C7—H7B	109.5
C3—C2—H2A	119.5	H7A—C7—H7B	109.5
C1—C2—H2A	119.5	O1—C7—H7C	109.5
C4—C3—C2	119.7 (4)	H7A—C7—H7C	109.5
C4—C3—H3A	120.1	H7B—C7—H7C	109.5
C2—C3—H3A	120.1		

### Hydrogen-bond geometry (Å, °)

**Table:**

*D*—H···*A*	*D*—H	H···*A*	*D*···*A*	*D*—H···*A*
N1—H1A···Br1	0.89	2.52	3.409 (3)	177
N1—H1B···Br1^i^	0.89	2.55	3.314 (3)	145
N1—H1B···Br1^ii^	0.89	3.00	3.430 (3)	112
N1—H1C···Br1^iii^	0.89	2.57	3.300 (3)	140

Symmetry codes: (i) −*x*, −*y*+1, −*z*; (ii) *x*−1/2, −*y*+1/2, −*z*; (iii) −*x*+1/2, *y*−1/2, *z*.
